# From Echo to Coronary Angiography: Optimizing Ischemia Evaluation Through Multimodal Imaging

**DOI:** 10.3390/medicina61122212

**Published:** 2025-12-15

**Authors:** Marija Babic, Lidija Mikic, Marko Ristic, Milorad Tesic, Snezana Tadic, Marija Bjelobrk, Dejana Popovic

**Affiliations:** 1 Department of Cardiology, University Clinical Centre of Serbia, 11000 Belgrade, Serbia; marijabb222@gmail.com (M.B.); marko.ris@live.com (M.R.); misa.tesic@gmail.com (M.T.); 2 Department of Cardiology, Hospital Center Zvezdara, 11120 Belgrade, Serbia; lidijamikic5@icloud.com; 3 Faculty of Medicine, University of Belgrade, 11000 Beograd, Serbia; 4 Department of Cardiology, Institute of Cardiovascular Diseases Vojvodina, 21204 Sremska Kamenica, Serbia; snezana.tadic@mf.uns.ac.rs (S.T.); bjelobrk.marija@gmail.com (M.B.); 5 Faculty of Medicine, University of Novi Sad, 21000 Novi Sad, Serbia; 6 Department of Cardiology, Mayo Clinic, Rochester, MN 55905, USA

**Keywords:** multimodal imaging, myocardial ischemia, noninvasive functional imaging, invasive coronary angiography

## Abstract

Multimodal imaging plays a central role in optimizing the evaluation and management of myocardial ischemia by leveraging the complementary strengths of echocardiography, cardiac magnetic resonance imaging (CMR), single photon emission computed tomography (SPECT), positron emission tomography (PET), and invasive coronary angiography (ICA). Noninvasive functional imaging is typically recommended for patients with intermediate to high pre-test probability of coronary artery disease, while coronary computed tomography angiography (CCTA) is preferred for low to intermediate risk. Stress echocardiography is valuable for detecting wall motion abnormalities and is particularly effective in multivessel or left main disease, where perfusion techniques may miss balanced ischemia. CMR offers high spatial resolution and quantitative assessment of myocardial blood flow (MBF), while SPECT and PET quantify ischemic burden, with PET providing superior accuracy for MBF and microvascular disease. ICA remains the gold standard for defining the presence, location, and severity of epicardial coronary stenosis. It is indicated when noninvasive imaging reveals high-risk features, when symptoms are refractory to medical therapy, or when noninvasive results are inconclusive. While ICA offers high spatial resolution, it alone cannot assess the hemodynamic significance of intermediate lesions, nor the coronary microvasculature. Adjunctive invasive hemodynamic and provocative coronary testing (e.g., Fractional Flow Reserve—FFR, invasive Coronary Flow Reserve—CFR, Index of Microcirculatory Resistance—IMR, acetylcholine test) provide essential insights, especially in ischemia with nonobstructive coronary arteries. Given its procedural risks, ICA should be reserved for cases where it will impact management. Intravascular imaging may be used to further characterize lesions. In summary, modality selection should be individualized based on patient characteristics, comorbidities, contraindications, and the need for anatomical versus physiological data. Integrating noninvasive and invasive modalities provides a comprehensive, patient-centered approach to ischemia evaluation.

## 1. Introduction

Coronary artery disease (CAD) remains a leading cause of morbidity and mortality worldwide [1]. Early and accurate detection of myocardial ischemia, the mismatch between myocardial oxygen supply and demand, is crucial for risk stratification, guiding revascularization decisions, and improving outcomes. Historically, imaging strategies have been either anatomical (defining coronary atherosclerosis) or functional (assessing the hemodynamic consequence of stenoses). However, the pathophysiology of CAD is far more complex, involving epicardial stenoses, microvascular dysfunction, plaque vulnerability, and myocardial tissue response. Recent guideline updates emphasize a tailored imaging pathway that takes into account pre-test probability, patient comorbidities, and imaging availability [2].

## 2. Objective

Despite the growing acknowledgements in pathophysiology of coronary circulation, we are still facing a common clinical approach to guide the decision making based on coronary angiogram alone. The aim of this article is to strengthen the statement that no single technique can provide the full spectrum of information about coronary circulation, and that angiographic appearance is not a reliable predictor of ischemia, especially in cases of intermediate lesions, diffuse disease, and older adults with calcifications. We want to emphasize the importance of combining different multimodal diagnostic tests in order to have a complete and comprehensive approach to ischemic heart disease.

## 3. Materials and Methods

### 3.1. Data Sources

We searched Pubmed, Google Schoolar, and Scopus Databases from inception to the present. The search terms included echocardiography, ICA, CT angiography, SPECT, PET, CMR as well as their stress modalities in relation to myocardial ischemia. Our focus was not on the statistical analysis but on synthesizing the available evidence and providing a comprehensive overview relevant to the multimodality assessment of coronary artery disease.

### 3.2. Inclusion Criteria

We included human studies investigating diagnostic modalities for quantitative and qualitative assessment of myocardial ischemia, and the data regarding the efficiency and safety of their use. We included review articles and different meta-analysis relevant to the above mentioned topics.

### 3.3. Exclusion Criteria

We excluded preclinical or animal studies, case reports and the articles evaluating diagnostic tools in patients with non-ischemic heart disease.

## 4. Rationale for a Multimodal Strategy

A key principle in contemporary imaging is that no single technique can provide the full spectrum of information—anatomical burden of disease, functional significance of lesions, myocardial perfusion, viability, left ventricular function, and microvascular integrity [3,4]. For example, a moderate stenosis on computed tomography angiography (CTA) may not cause ischemia, whereas microvascular dysfunction may produce symptoms despite angiographically normal epicardial arteries. The growing concept in ischemic heart disease is that obstructive epicardial coronary disease represents just the tip of the iceberg, and that 50–70% females and 30–50% males with anginal symptoms have in fact microvascular dysfunction, that can be addressed as a spectrum of different pathophysiological changes called ANOCA or INOCA (angina and ischemia with no obstructive coronary artery disease, respectively). The only way of diagnosing microvascular dysfunction is by functional testing [5]. Thus, integration of anatomical and functional data improves diagnostic accuracy and guides management. Multimodal imaging plays a central role in optimizing the evaluation and management of myocardial ischemia by leveraging the complementary strengths of echocardiography, CTA, single-photon emission computed tomography (SPECT), positron emission tomography (PET), cardiac magnetic resonance imaging (CMR), and invasive coronary angiography (ICA) [6].

When choosing an appropriate diagnostic method in evaluating ischemic heart disease important factors are resource utilization as well as good patient selection. Imaging should be chosen with the aim of influencing clinical management in a cost-effective manner. As recent Appropriate Use Criteria emphasize, modality choice must account for the clinical scenario, risk profile, and availability of equipment and expertise [6]. Standard exercise stress test (EST) is a widely available, inexpensive, radiation free method, but due to low sensitivity (66%) and specificity (61%), it is not considered as an appropriate choice for the clinical evaluation of patients with suspected CAD [7,8]. The advantages and limitations of different diagnostic methods for assessing myocardial ischemia are presented in Table 1.

In this context, a pragmatic algorithm has emerged: patients with low to intermediate pre-test probability (typically <50%) are best served by anatomical imaging (CTA) to exclude obstructive CAD; those with intermediate to high probability undergo functional stress imaging to detect inducible ischemia; those with high-risk features or inconclusive non-invasive testing proceed to ICA [2,3,4,6].

**Figure 1:**
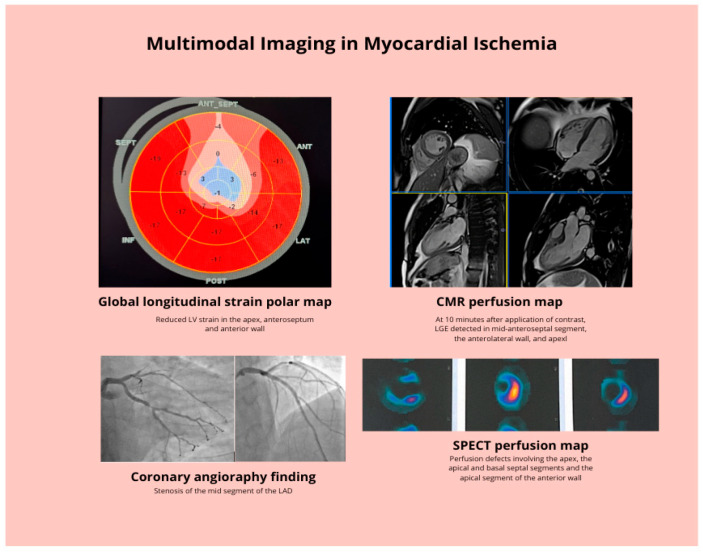
Multimodal Imaging in Myocardial Ischemia.

This review aims to provide a comprehensive, modality-by-modality discussion of current imaging techniques in the assessment of myocardial ischemia and coronary microvascular disease. By presenting a structured summary of each modality—echocardiography, CTA, SPECT, PET, CMR, and ICA—followed by considerations for an integrated approach, we aim to support clinicians and imaging specialists in selecting the appropriate test or combination of tests for individual patients. In doing so, we emphasize that the value of modern imaging lies not merely in acquiring images, but in using them thoughtfully to guide patient-centered decision making Figure 1.

## 5. Echocardiography in the Assessment of Myocardial Ischemia

Echocardiography is a widely accessible imaging modality for evaluating myocardial ischemia, capable of reliably detecting regional wall motion abnormalities (RWMA) such as reduced systolic thickening, hypokinesia, akinesia, and dyskinesia of the left ventricle, which reflect the downstream mechanical consequences of myocardial ischemia [9]. It plays an essential role in both the diagnosis and longitudinal monitoring of ischemic left ventricular remodeling. Among imaging techniques, three-dimensional echocardiography, besides CMR imaging, provides the most accurate volumetric assessment of cardiac chambers [9].

Beyond conventional two-dimensional assessment of systolic function through left ventricular ejection fraction, modern echocardiography incorporates two-dimensional speckle-tracking echocardiography (STE) to quantify global and regional longitudinal, circumferential, and radial strain [10,11]. Longitudinal strain is particularly sensitive to myocardial ischemia and often reveals subclinical systolic dysfunction before any overt ventricular remodeling or reduction in left ventricular ejection fraction becomes apparent [10] Variations in strain curves, especially in peak systolic strain, reflect regional disparities in myocardial deformation. Absent, reduced, or delayed peak systolic strain indicates impaired contractility and altered timing of myocardial shortening [10,11].

Standard echocardiography remains a cornerstone of evaluation in all patients with ischemic heart disease. When conventional findings are inconclusive, global longitudinal strain of the left ventricle should be assessed using STE [10,11]. In addition to systolic function, two-dimensional echocardiography allows evaluation of left ventricular diastolic function and indexed left atrial volume. STE further provides comprehensive assessment of left atrial reservoir, conduit, and contractile function [11]. This integrated approach enables earlier detection of pathophysiological mechanisms underlying heart failure with preserved ejection fraction, particularly in patients with coronary microvascular disease (CMD) or angina with non-obstructive coronary arteries (ANOCA) [11]. However, it is worth mentioning that wall motion stress testing has limited sensitivity for single-vessel disease and may be falsely negative in balanced ischemia [8]. 

Contrast echocardiography enhances the diagnostic performance of standard echocardiography by improving endocardial border delineation, especially in patients with suboptimal acoustic windows. The intravenous administration of microbubble agents enables more accurate assessment of cardiac volumes, ejection fraction, and regional wall motion, thereby increasing the sensitivity and specificity for detecting myocardial ischemia [8,9,10]. Similarly, in stress echocardiography, contrast enhancement improves visualization of subtle RWMA and allows quantitative perfusion imaging, offering additional information on myocardial blood flow and microvascular integrity [12,13]. This method is particularly valuable in critically ill or obese patients or when image quality is limited by lung interference or chest wall anatomy [14]. Moreover, contrast perfusion imaging can detect regions of myocardial hypoperfusion that may not be apparent on standard two-dimensional imaging, allowing earlier identification of ischemic myocardium and guiding decisions regarding revascularization [12,14]. Overall, this is a safe, minimally invasive, and highly informative method adjunct to both resting and stress echocardiography, complementing STE and conventional assessment in the comprehensive evaluation of ischemic heart disease and coronary microvascular dysfunction [10,11,12,13,14].

For the non-invasive functional assessment of CAD, stress echocardiography remains a cost-effective, and widely available modality [15]. Stress echocardiography is generally considered a safe method, but it also carries risk of serious complications, such as life threatening arrhythmias, acute myocardial infarction and cardiac rupture [16]. According to the Spanish study during a 21-year period and 19239 treadmill tests performed, the risk of complications was approximately 0.02–0.04% [17]. Modern stress echocardiography protocols, particularly the ABCDE framework, allow simultaneous evaluation of congestion, contractile and chronotropic reserve, and microcirculatory function [11,15]. Integration of STE into these protocols enhances both sensitivity and specificity for detecting ischemia [10,11]. Stress echocardiography can either increase myocardial oxygen demand, via exercise or pharmacological dobutamine stress echocardiography, or induce coronary vasodilation using agents such as dipyridamole or adenosine [12,18]. In the presence of functionally significant epicardial stenosis, increased myocardial demand leads to worsening RWMA, which can be quantified using the wall motion score index [12,18]. Ischemia may also manifest through anginal symptoms, electrocardiographic changes, or hypotension, suggestive of left main or multivessel coronary disease [12]. Stress echocardiography is particularly useful in patients with moderate-to-high pre-test probability of obstructive CAD, suspected stent or graft restenosis, and for risk stratification regarding major adverse cardiovascular events (MACE) [12,14,19].

Low-dose dobutamine stress echocardiography is highly effective for assessing myocardial viability. Improvement in wall motion and regional longitudinal strain during low-dose infusion indicates viable myocardium, especially in hibernating regions, which can be visualized using STE-derived polar maps. The biphasic response, characterized by improvement at low dose followed by deterioration at peak dose, identifies chronically ischemic myocardium that is likely to benefit from revascularization [20,21]. 

Vasodilatory stress echocardiography operates through a distinct mechanism. Dipyridamole induces dilation of both epicardial arteries and the coronary microcirculation, and its prolonged infusion makes it suitable for assessing left ventricular kinetics [18]. All vasodilator agents also permit evaluation of coronary flow reserve (CFR) by Doppler imaging of epicardial coronary arteries. CFR is defined as the ratio of myocardial blood flow velocity after stress to that at rest (normal value ≥ 2) [18,22]. CFR measurement provides insight into epicardial stenosis, CMD, and prognosis across various cardiac and systemic diseases [18,22]. Echocardiographic CFR correlates closely with invasive measures such as fractional flow reserve (FFR) and instantaneous wave-free ratio (iFR), and it can be assessed in all three major coronary arteries [6,22].

Coronary microvascular disease involves both structural and functional impairment of the small coronary vessels (diameter < 400 μm), resulting in reduced CFR and abnormal myocardial perfusion either at rest or under stress [22,23]. Non-invasive assessment typically targets the distal left anterior descending artery (LAD) using optimized transducer positioning, color Doppler imaging, and pulse-wave sampling [22,23]. Stress-induced distal LAD CFR correlates well with invasive measurements of microcirculatory resistance, while elevated baseline coronary flow velocity may indicate the presence of CMD [9,22]. Within the ABCDE stress echocardiography framework, CFR assessment complements RWMA evaluation and enhances risk stratification for MACE, particularly in patients with chronic coronary syndrome [18,22]. While having in mind all benefits of CFR, we must conclude that it still is technically demanding, time consuming, operator dependent technique, and mostly limited to the LAD evaluation [18]. 

Technological advances promise further refinement of echocardiographic ischemia assessment. Automated strain analysis, 3D-speckle tracking, myocardial work indices (pressure-strain loops), and artificial intelligence (AI)-assisted image acquisition and interpretation hold potential to reduce operator-dependence and improve reproducibility. Continued integration of echocardiographic functional parameters into risk stratification models and comparative validation against PET/CT and invasive microvascular measures will be critical [15,19]. 

### Practical Considerations and Integration of Echocardiography into Multimodal Strategy

Echocardiography is the first-line imaging modality for suspected ischemic heart disease because it is widely available, radiation-free, and provides real-time assessment of cardiac structure, valvular function, and hemodynamics. Although it cannot directly visualize coronary anatomy, it serves as a key functional gatekeeper, particularly when anatomical imaging is inconclusive. Stress echocardiography is valuable in patients with intermediate pre-test probability of obstructive CAD to guide management decisions. However, its accuracy can be limited by suboptimal acoustic windows, obesity, lung disease, or chest wall deformities, as well as operator dependency and potential underestimation of balanced ischemia in multivessel disease [15,19]. Coronary flow reserve measurement, though informative, remains technically demanding and typically limited to the distal LAD [23].

## 6. Coronary Computed Tomography Angiography in the Assessment of Myocardial Ischemia

Coronary CT angiography (CCTA) has become a central tool in the assessment of CAD, supported by growing evidence for its integration into ischemia assessment. Beyond its established role in anatomical visualization, recent advances have enabled CCTA to provide insights into plaque biology, risk stratification, and functional assessment through CT-derived techniques such as CT-fractional flow reserve (CT-FFR) and CT perfusion (CTP).

CCTA allows direct visualization of the coronary lumen and wall, enabling non-invasive detection of coronary stenosis and plaque burden [24]. CCTA identifies high-risk plaque features, such as low-attenuation plaque, positive remodeling, and spotty calcification, linked with future acute coronary events. Large trials including SCOT-HEART and PROMISE [25,26], demonstrated that CCTA not only improves diagnostic accuracy but also provides incremental prognostic value, influencing downstream management and reducing hard cardiovascular events [27,28].

On a per-segment basis, CCTA predicts significant stenosis (≥50%) with a sensitivity of 89%, specificity of 96%, positive predictive value of 78%, and negative predictive value of 98% [24]. Its high negative predictive value makes CCTA particularly valuable for excluding obstructive CAD in low- or intermediate-risk patients and acting as a gatekeeper for coronary angiography [24].

CT perfusion evaluates first-pass myocardial perfusion at rest and stress. Pharmacologic hyperemia (adenosine, dipyridamole, or regadenoson) allows the detection of ischemia-related perfusion defects.

Overall, CTP offers high spatial resolution, direct visualization of perfusion defects, and the ability to evaluate ischemia even in patients with heavy calcifications or stents, situations where CT-FFR is limited. However, the method requires advanced scanner technology and expertise, involves higher radiation and contrast exposure, and remains less validated in patients post-CABG. Static CTP provides qualitative perfusion data, whereas dynamic CTP enables quantitative assessment of myocardial blood flow, independent of scan timing [19,29]. Several pivotal clinical studies have validated the diagnostic role of CTP. The CORE320 trial demonstrated that combining CCTA with CTP significantly improved specificity and diagnostic accuracy compared with CCTA alone, and outperformed SPECT when referenced against invasive angiography with FFR [25,30]. The PERFECTION study showed that CTP and CT-FFR offer comparable diagnostic performance, both significantly improving the specificity of CCTA in patients with suspected CAD [31]. The PACIFIC trial, which compared CTP, CT-FFR, PET, and SPECT against invasive FFR, confirmed that while PET provided the highest overall accuracy, both CTP and CT-FFR performed better than SPECT and added robust functional information beyond CCTA alone [32]. Earlier feasibility studies, including those by Bamberg et al. and Rossi et al., further established the role of dynamic CTP in quantifying myocardial blood flow and improving ischemia detection [33,34]. 

CT-Derived Fractional Flow Reserve estimates lesion-specific ischemia by applying computational fluid dynamics or machine learning to CCTA datasets. It simulates invasive FFR without catheterization. CT-FFR uses patient-specific coronary anatomy obtained from CCTA and applies computational modeling to simulate blood flow and pressure under conditions of maximal hyperemia. The technique outputs a fractional flow reserve value, defined as the ratio of distal coronary pressure to aortic pressure, for each coronary lesion. A value ≤ 0.80 indicates hemodynamically significant stenosis. Clinically, CT-FFR is particularly valuable in the evaluation of intermediate coronary stenoses (40–70%) detected on CCTA, where the functional significance of the lesion is uncertain. By providing a noninvasive physiological assessment, CT-FFR reduces unnecessary ICA and supports decision-making regarding revascularization, helping to identify which lesions are likely to benefit from percutaneous coronary intervention (PCI) or coronary artery bypass grafting (CABG). Importantly, an abnormal CT-FFR value not only correlates with ischemia-producing lesions but also serves as a predictor of adverse cardiovascular outcomes, enhancing its role in patient risk stratification and management planning. CT-FFR improves specificity of CCTA, guides revascularization decisions, and adds prognostic value, although availability and image quality remain limitations [29]. 

Large multicenter studies have confirmed the diagnostic and clinical value of CT-FFR. The NXT trial demonstrated superior diagnostic accuracy of CT-FFR compared with CCTA alone (81% vs. 53%) [35]. In the PLATFORM study, CT-FFR reduced unnecessary ICA by 61% without increasing adverse events [22,36]. The ADVANCE registry, which included more than 5000 patients across 38 countries, confirmed the real-world feasibility and prognostic value of CT-FFR [33]. Importantly, an abnormal CT-FFR not only correlates with ischemia-producing lesions but also predicts adverse cardiovascular outcomes, thereby enhancing its role in risk stratification and management planning.

Coronary CT angiography and CT-derived functional techniques are evolving rapidly toward comprehensive, noninvasive assessment of CAD. Advances in artificial intelligence and machine learning promise to automate image analysis, plaque characterization, and CT-FFR computation, reducing operator dependency and enabling near real-time functional assessment. Dynamic CTP is likely to become increasingly quantitative, providing absolute myocardial blood flow measurements and improving the evaluation of ischemia in complex lesions. Technological innovations, such as high-speed gantry rotation, wide detectors, and iterative reconstruction, will further improve image quality while minimizing radiation exposure. Integration with other imaging modalities, including PET and CMR, may facilitate hybrid approaches that combine anatomical, functional, and metabolic information for precise risk stratification [37]. 

### Practical Considerations and Integration of Coronary Computed Tomography Angiography into Multimodal Strategy

Despite its strengths, CCTA is limited in determining the functional significance of stenosis. There is no linear correlation between anatomical narrowing and ischemia. Only about 49% of stenoses ≥ 50% on CCTA correspond to functionally significant lesions defined by invasive FFR < 0.75 [27]. Causes of discordance include visual overestimation and heavy calcification, most commonly seen in patients with chronic kidney disease or chronic inflammation [38,39]. Therefore, lesions of moderate severity (50–69%) often require further functional assessment, either with noninvasive stress imaging or invasive FFR. This limitation has driven the development of CT-derived techniques to bridge the anatomical-functional gap [27]. 

CCTA is now considered a Class I recommendation in the evaluation of both acute and chronic coronary syndromes by the ESC, NICE, and the 2021 ACC/AHA Chest Pain Guideline [5,19,37]. According to the 2024 ESC Guidelines for the management of chronic coronary syndromes, in individuals with suspected chronic coronary syndromes and low or moderate (>5–50%) pre-test likelihood of obstructive CAD, CCTA is recommended to diagnose obstructive CAD and to estimate the risk of MACE (class I, level A). CCTA is recommended in individuals with low or moderate (>5–50%) pre-test likelihood of obstructive CAD to refine diagnosis if another non-invasive test is non-diagnostic (class I, level A). CCTA is not recommended in patients with severe renal failure (eGFR < 30 mL/min/1.73 m^2^), decompensated heart failure, extensive coronary calcification, fast irregular heart rate, severe obesity, inability to cooperate with breath-hold commands, or any other conditions that can make obtaining good imaging quality unlikely (class III, level C) [7]. 

CCTA has evolved from a purely anatomical tool into a cornerstone of multimodality imaging in CAD. CT-derived functional techniques, particularly CT-FFR and CTP, enhance diagnostic accuracy, provide critical prognostic information, and reduce unnecessary ICA. Optimal clinical practice requires a tailored multimodality approach, based on patient characteristics, clinical scenario, and resource availability.

## 7. SPECT and PET in the Assessment of Myocardial Ischemia

Single-photon emission computed tomography—SPECT and positron emission tomography—PET are functional nuclear imaging techniques in which radiotracers are administered to patients and accumulate in specific tissues based on molecular and physiological characteristics [40]. These methods enable assessment of organ function at the cellular and molecular level. Radiotracers are molecules containing unstable nuclides that emit energy in the form of gamma photons or positrons to reach stability [40,41]. In SPECT, the emission of a single gamma photon allows localization of the radionuclide, whereas in PET, a positron interacts with an electron, producing two gamma photons detected in coincidence by external gamma cameras. Reconstruction algorithms combined with attenuation correction from a simultaneous CT scan provide precise anatomical and functional images [42].

SPECT and PET are widely used in the evaluation of CAD and coronary microcirculation [7]. In clinical practice, myocardial perfusion is assessed both at rest and during stress to identify areas of inducible ischemia that can guide revascularization strategies [43]. Stress is induced either by exercise or pharmacological agents such as dobutamine, dipyridamole, adenosine, or regadenoson [42]. The most commonly used radiotracers target Na^+^/K^+^-ATPase or mitochondria in viable cardiomyocytes [41]. Radiotracer uptake is proportional to regional myocardial blood flow (MBF), with reduced uptake during stress indicating hypoperfusion and reduced uptake during both stress and rest suggesting myocardial scar [44].

Although both modalities are based on similar nuclear principles, they differ in clinical application and technical performance. Traditional SPECT is more widely available and cost-effective, but it involves longer acquisition times, higher radiation exposure, and lower spatial resolution than PET [43]. Perfusion imaging in SPECT is based on relative comparisons of signal intensity across the 17 myocardial segments, which can result in false-negative findings in multivessel or balanced ischemia [43]. In contrast, PET provides superior spatial and temporal resolution, faster acquisition, and more accurate attenuation correction. These features enable dynamic imaging and quantitative assessment of MBF and myocardial flow reserve (MFR) [29]. Quantitative parameters derived from PET hold both diagnostic and prognostic value [45]. PET also offers a lower radiation burden but remains less accessible and more expensive [43,44].

The most commonly used radiotracers for SPECT include technetium-99m tetrofosmin, which targets mitochondria, and thallium-201, which binds to Na^+^/K^+^-ATPase. Due to its higher radiation exposure, thallium-201 is now less frequently used. PET tracers include rubidium-82 chloride (Na^+^/K^+^-ATPase target), nitrogen-13 ammonia (glutamine synthetase target), and oxygen-15 water, which diffuses freely across membranes. Novel tracers such as fluorine-18 flurpiridaz, which targets mitochondrial complex I, are under active development and show promise for improved image quality and workflow efficiency [29].

Nuclear imaging also enables metabolic evaluation of the myocardium. During ischemia, cardiomyocytes shift from fatty acid to glucose metabolism, a process that can be visualized using 18F-fluorodeoxyglucose (FDG) [44]. Dual-tracer techniques such as 99mTc-tetrofosmin and 123I-BMIPP in SPECT or 18FDG with 13N-ammonia or 82Rb in PET allow simultaneous evaluation of perfusion and metabolism. Combined reductions in both perfusion and metabolism indicate nonviable myocardium, while preserved metabolism despite reduced perfusion suggests hibernating myocardium, and preserved perfusion with impaired metabolism indicates stunned myocardium following reperfusion. Both hibernating and stunned myocardium are considered viable and may benefit from revascularization [44,46,47].

Recent advances in nuclear cardiology have focused on improving image quality, enabling absolute flow quantification, and enhancing diagnostic accuracy through hardware and software innovation. In SPECT, the introduction of cadmium-zinc-telluride semiconductor detectors has led to significant progress. These detectors enable shorter acquisition times, improved spatial and energy resolution, and potential for quantitative MBF assessment comparable to PET [46].

Quantification of MBF and MFR has become increasingly important in risk stratification. Normal stress MBF and MFR values are associated with a high negative predictive value for excluding high-risk obstructive CAD [45]. Dietz et al. demonstrated that impaired global stress MBF, global MFR, and regional myocardial flow capacity are strong predictors of cardiovascular events, with global stress MBF being the most robust independent predictor of MACE [48]. PET/CT-based MACE-Revasc risk scores have been developed to further refine prognostic prediction at 90 days and one year [49]. As the understanding of chronic coronary syndromes expands from epicardial to microvascular dysfunction, PET has become a central tool for evaluating coronary microcirculation. A systematic review and meta-analysis showed that in patients with non-obstructive CAD, PET detected microvascular dysfunction in 54% of cases [50].

The future of both SPECT and PET also lies in the development of new radiotracers targeting specific molecular pathways and the incorporation of AI and deep learning for improved image reconstruction, automated quantification, and prognostic modeling [41,51]. These innovations are expected to enhance the clinical utility of nuclear cardiology and contribute to precision medicine approaches in CAD management.

### Practical Considerations and Integration of SPECT and PET into Multimodal Strategy

In clinical practice, SPECT and PET are central to the diagnostic work-up of patients with suspected or established CAD. According to the 2024 European Society of Cardiology Guidelines for the Management of Chronic Coronary Syndromes, stress SPECT, and preferably PET, is recommended (Class I, Level of Evidence B) for patients with moderate-to-high (15–85%) pre-test likelihood of obstructive CAD to diagnose and quantify myocardial ischemia and/or scar, estimate the risk of MACE, and quantify MBF [5]. Measurement of coronary artery calcium score from unenhanced CT imaging used for attenuation correction is also recommended to improve detection of both obstructive and non-obstructive CAD (IB) [7]. Furthermore, in persistently symptomatic patients with documented or suspected angina or ischemia with non-obstructive coronary arteries (ANOCA/INOCA), PET may be considered for the non-invasive assessment of CFR (Class IIb, Level B) [7].

From a practical perspective, both modalities require careful selection of stress protocols, which may include exercise or pharmacologic agents such as dobutamine, dipyridamole, adenosine, or regadenoson [42]. SPECT remains semi-quantitative and prone to false negatives in balanced ischemia, and false positives due to poor spatial resolution and attenuation artifacts (left bundle branch block with septal motion abnormality can mimic septal perfusion defect; breast, diaphragm, or hepatobiliary tract interposition can mimic anterior or inferior wall perfusion defects) [44].

On the contrary, PET enables absolute quantification of MBF and MFR with higher accuracy. PET typically involves lower radiation exposure and shorter acquisition times but remains limited by higher cost and reduced availability [43,44]. However, in specific clinical scenarios (e.g., severe left ventricular hypertrophy, diffuse coronary disease), quantification of MBF can be challenging [48].

Despite these differences, both techniques remain indispensable for comprehensive functional evaluation of the myocardium. The integration of quantitative imaging, new detector technologies, and AI-driven analysis is expected to further refine diagnosis, prognosis, and individualized management of patients with CAD.

## 8. Cardiac Magnetic Resonance Imaging in the Assessment of Myocardial Ischemia

Cardiovascular magnetic resonance imaging is an advanced non-invasive modality that provides comprehensive evaluation of cardiac anatomy, function, perfusion, and tissue composition without ionizing radiation. Its high spatial and temporal resolution, coupled with accumulation of gadolinium contrast, allows for precise assessment of the myocardium, and coronary perfusion making it a cornerstone tool in the detection and characterization of myocardial ischemia [52,53]. The general approach to ischemia assessment with CMR relies on a multiparametric protocol that integrates structural, functional, and tissue-specific sequences. Cine MRI sequences enable accurate quantification of ventricular volumes and wall motion throughout the cardiac cycle [54]. The standard protocol for stress MRI is based on using gadolinium contrast for tracing the blood flow and subsequent myocardial perfusion after the injection of a vasodilator and the onset of the hyperemia. The series of T1 weighted images are acquired a few cardiac cycles after IV contrast application. The gadolinium contrast is distributed through the epicardial coronary vessels into the microcirculation and myocardial tissue. Normally perfused myocardium will be seen as higher intensity of T1 signal. The perfusion defect will appear as hypointensity in the area of coronary artery disease. After 10 to 15 min, resting images are acquired. Then, five minutes later, which is the time allowing healthy myocardium to washout the contrast, images are acquired again, but this time to evaluate the pattern of late gadolinium enhancement. In the areas with more extracellular volume most often due to fibrosis, the washout of gadolinium is slower, thus it accumulates. Comparing perfusion defects to myocardial tissue viability can guide clinical decision on revascularization. Defects in the perfusion of viable myocardium indicate benefit from revascularization, and perfusion defect compatible with LGE pattern, indicate the zone of infarction [55]. In rare cases, there can be evidence of myocardial infarction without apparent perfusion defects, which can be related to complex pathophysiology of MINOCA [56]. That being said, late gadolinium enhancement (LGE) imaging is the gold standard for assessing myocardial infarction and viability, exploiting the differential accumulation of gadolinium contrast in scarred versus healthy tissue to distinguish viable from non-viable myocardium and guide revascularization decisions [52]. In the acute phase of myocardial infarction, CMR can delineate myocardial edema via T2 mapping, identifying microvascular obstruction and intramyocardial hemorrhage that may persist despite reperfusion, and evaluating tissue heterogeneity through native T1 and T2 mapping [57,58,59]. Increased myocardial water content prolongs both T1 and T2 relaxation times, while areas of microvascular obstruction demonstrate reduced T1 relative to the surrounding infarct but remain elevated compared with normal myocardium [57,60,61]. Quantification of the extracellular volume fraction, derived from pre- and post-contrast T1 values corrected for hematocrit, provides additional information on myocardial fibrosis and remodeling. Elevated extracellular volume may be observed even in regions appearing normal on conventional imaging, suggesting subclinical injury [62]. During the chronic phase, CMR enables precise quantification of scar burden, identification of post-infarct complications such as aneurysm or thrombus, and evaluation of left ventricular remodeling, which are critical for prognosis and management [52,63]. T1 mapping correlates strongly with LGE in quantifying chronic infarct size, while extracellular volume fraction values exceeding 42% indicate mature scar tissue [61,64]. Although myocardial wall thinning is related to myocardial scar and LGE accumulation, in the study from Shah and authors, 18% of 1055 patients with CAD had regional wall thinning with little or no scarring. In this study population, the absence of LGE was associated with better prognosis and with improved contractility after revascularization [65]. CMR is superior to SPECT in differentiating balanced ischemia, as it has higher spatial resolution, allowing for more detailed perfusion analysis, without the impact of surrounding tissue artifacts, which are common in SPECT. Contrary to SPECT perfusion analysis that requires a portion of healthy myocardium for comparison, CMR allows for the analysis of all segments and layers of myocardium (subendocardial vs. transmural ischemia) [55].

Future advancements are expanding the diagnostic and prognostic potential of CMR in ischemic myocardial disease. Quantitative stress perfusion CMR is rapidly evolving, enabling absolute measurement of MBF and perfusion reserve to improve detection of coronary microvascular dysfunction and refine risk stratification, particularly in patients with INOCA [3,66]. The myocardial perfusion reserve (MPR), defined as the ratio of blood flow during stress to rest, serves as a key physiological marker, with values below 1.5 consistent with ischemia [52]. Emerging parametric mapping techniques, including T1, T2, and ECV mapping, as well as novel sequences such as MR fingerprinting and four-dimensional (4D) flow, allow earlier clinical adoption and enhance the role of CMR in precision cardiovascular imaging [67,68,69].

### Practical Considerations and Integration of CMR into Multimodal Strategy

From a practical perspective, contemporary guidelines endorse CMR as a first-line or complementary imaging modality for the evaluation of myocardial ischemia, viability, and infarction. The American College of Cardiology, American Heart Association, and American Society of Echocardiography, in collaboration with other societies, recommend stress perfusion CMR for patients with chest pain and intermediate-to-high risk of CAD, particularly when prior noninvasive tests are inconclusive [66]. LGE imaging remains the reference standard for differentiating viable from non-viable myocardium, guiding revascularization, and informing prognostic assessment [3,66]. CMR also provides essential information for patients with ischemic cardiomyopathy, enabling quantification of scar burden and detection of post-infarct complications such as left ventricular thrombus and pericarditis [66,67,68,69,70,71,72,73]. Moreover, it is increasingly used in patients with INOCA and microvascular angina, where it clarifies the etiology of ischemia and informs management [69,73,74]. Guidelines support these applications, citing the modality’s high diagnostic accuracy, reproducibility, and prognostic value [3,75].

Despite these advantages, several limitations restrict the widespread use of CMR. The modality requires costly and technically demanding equipment, extensive operator expertise, and substantial acquisition and post-processing time [75]. Its availability remains limited in many healthcare settings, and certain patients with older pacemakers or defibrillators remain ineligible, although MR-conditional systems have expanded safety [76]. There is an established protocol for patients with cardiac implantable electronic devices (CIEDs), which includes device reprogramming prior to stress imaging and subsequent restoration of baseline settings after the procedure. In such cases, increased awareness and monitoring during the examination are required to ensure patient safety [77,78]. Artifacts from metallic implants or stents, CIED and breath-hold requirements can affect image quality, particularly in patients with pulmonary disease. Compared to other modalities, such as echocardiography, CT, or nuclear techniques, CMR offers unique advantages including superior spatial resolution, absence of radiation, and simultaneous assessment of structure, function, and tissue composition [59].

## 9. Coronary Angiography and Intracoronary Imaging

Invasive assessment of coronary artery anatomy has long been recognized as fundamental for the detection, diagnosis, and treatment of ischemic heart disease, ever since autopsy findings in patients presenting with acute myocardial infarction revealed coronary artery occlusion as the key causal mechanism underlying pathophysiological disturbances and sudden cardiac death [79]. Over the years, interventional cardiology has advanced remarkably, surpassing cardiac surgery in many aspects by offering minimally invasive, safer, and highly effective treatment options for varying degrees of CAD [80]. Continuous innovations in biomedical engineering have transformed coronary angiography from a purely diagnostic technique into a therapeutic intervention, with the development of multiple generations of stents designed to maintain long-term vessel patency and improve myocardial perfusion [81,82]. Guided by the principles of personalized and evidence-based medicine, both acute and chronic coronary syndromes represent distinct clinical entities in which the integration of clinical and interventional cardiology remains essential for optimal decision-making and outcomes. Furthermore, the introduction of intracoronary imaging and physiological measurements, such as indices of flow reserve and vascular resistance, has enabled more accurate procedural planning and tailored interventional strategies. Among these, intravascular ultrasound (IVUS) and optical coherence tomography (OCT) continue to evolve, with ongoing improvements in catheter design and imaging software enhancing spatial resolution and diagnostic precision [83].

As complex percutaneous coronary intervention (PCI) techniques evolved, the need arose to better understand the transition from angiographic findings to optimal lesion preparation, particularly in cases of calcified stenoses, suboptimal balloon expansion, and resistant plaques. Many patients undergo repeat PCI procedures due to disease progression or in-stent restenosis, necessitating deeper insight into the structural and functional consequences of prior interventions. The development of intracoronary catheters equipped with phased-array ultrasound transducers enabled real-time imaging of the coronary vessel wall, providing detailed cross-sectional visualization of arterial layers [83,84]. These rapidly rotating probes, operating at variable pullback speeds, generate two-dimensional images that delineate fibrotic and calcified regions as hyperechogenic areas, while lipid-rich components and hematomas appear darker due to reduced sound absorption [84]. The imaging console allows for precise measurements of lumen and wall dimensions, calculation of vessel and plaque areas, and selection of appropriate stent size and expansion strategy, thereby facilitating lesion preparation and plaque modification prior to stent implantation. The use of IVUS has been shown to reduce contrast volume during complex PCI [85], and to improve both procedural outcomes and long-term prognosis [86,87,88]. The European Bifurcation Club consensus document highlights the utility of IVUS throughout every stage of left main disease management, from revascularization decision-making and PCI strategy planning to stent landing zone selection and optimization [89]. One of the most common applications of IVUS is in the management of calcified lesions, where it allows calculation of a calcium score based on imaging features, guiding the need for atherectomy and assessing the risk of stent underexpansion [90]. According to the Society for Cardiovascular Angiography and Interventions consensus on calcified lesion management, IVUS can quantify calcium arc and length, while only OCT can precisely measure calcium thickness [91]. IVUS is also invaluable in managing PCI complications, including ostial dissections, stent strut protrusions, crushed stents, vessel perforations, and side branch occlusions [92,93]. It provides critical guidance during chronic total occlusion interventions by confirming guidewire position within the true lumen [94,95]. Additionally, IVUS serves an important diagnostic role in detecting post-transplant cardiac allograft vasculopathy and is recommended as a Class IIa indication in the 2023 International Society for Heart and Lung Transplantation (ISHLT) Guidelines [96,97].

A fiber-optic frequency-domain imaging system utilizing near-infrared light enables high-resolution visualization of vascular structures, delineating the morphological layers of the vessel wall with exceptional precision and safety [84,94,97]. Compared with IVUS, OCT offers superior spatial resolution, faster image acquisition, and software-assisted three-dimensional vessel reconstruction [84,98]. However, IVUS provides greater tissue penetration and does not require contrast injection to eliminate imaging artifacts, a necessary step in OCT imaging because red blood cells scatter infrared light and obscure visualization [84,94]. When performing OCT, adherence to the “4Ps” principle, position, purge, puff, and pullback, is essential for optimal image acquisition [97]. Several mnemonic approaches guide interpretation and procedural planning using OCT. For instance, when assessing stent underexpansion in calcified lesions, the “rule of 5 s” scoring system is applied: maximum calcium thickness > 0.5 mm, contiguous calcium length > 5 mm, and calcium arc > 50% of the vessel circumference each contribute one point. A total score ≤ 2 generally indicates that additional plaque modification techniques, such as rotational atherectomy or intravascular lithotripsy, are not required [97]. Furthermore, the “MLD MAX” algorithm has been proposed to standardize OCT-guided PCI. The first component, MLD (Morphology, Length, and Diameter), addresses critical pre-stenting factors, including plaque characterization and selection of appropriate stent dimensions, while *MAX* (Medial dissection, Apposition, and eXpansion) focuses on post-stenting optimization and complication prevention [98,99]. OCT provides detailed tissue characterization, distinguishing platelet-rich from white thrombi, lipid from fibrous plaques, and identifying high-risk lesions responsible for acute coronary syndromes [84,97]. Beyond plaque rupture, OCT can also detect less frequent but clinically important causes of acute coronary syndrome, such as spontaneous coronary artery dissection or vasospasm [100]. Accurate length assessment ensures safe stent landing zones and selection of appropriate stent and balloon sizes [99]. In the post-procedural phase, OCT allows early detection of stent-related complications, including medial dissection, malapposition, and underexpansion, as well as late events such as neointimal hyperplasia and neoatherosclerosis, which contribute to in-stent restenosis [101].

Evidence from large registries, randomized controlled trials (RCTs), and meta-analyses increasingly supports the clinical utility of OCT, demonstrating non-inferiority to IVUS and, in some cases, superior performance in achieving key interventional endpoints [97]. The Pan-London PCI registry (2005–2015) showed that OCT-guided PCI, used in 1.3% of patients, was associated with lower mortality rates compared with IVUS- (12.6%) and angiography-guided interventions (7.7% vs. 12.2% vs. 15.7%, respectively). OCT-guided procedures also achieved higher procedural success, longer mean stent length, and lower in-hospital MACE, a composite of all-cause mortality, myocardial infarction, stroke, and repeat PCI [102]. Similarly, the OCCUPI trial from South Korea reported a significantly lower incidence of MACE, including cardiac death, MI, stent thrombosis, and ischemia-driven target vessel revascularization, with OCT-guided PCI compared with angiography-guided PCI using everolimus-eluting stents [103]. The OCTOBER trial further demonstrated that OCT-guided PCI for complex bifurcation lesions significantly reduced the composite endpoint of cardiac death, target-lesion MI, or ischemia-driven target-lesion revascularization at a median two-year follow-up [104].

Emerging hybrid imaging techniques represent the next frontier in multimodality intravascular imaging, integrating OCT or IVUS with coronary physiology tools to provide a comprehensive assessment of both structure and function [95]. Near-infrared spectroscopy (NIRS) can identify lipid-rich plaques by quantifying their cholesterol content through the lipid-core burden index, where elevated values in non-culprit lesions have been associated with an increased risk of future cardiovascular events [84,105]. NIRS can be fused with both OCT and IVUS systems, as well as with fluorescence imaging modalities, thereby enhancing plaque characterization and overcoming the limitations inherent to individual intravascular imaging catheters [100,106].

Fractional flow reserve—FFR, instantaneous wave-free ratio—iFR, and the index of microcirculatory resistance—IMR are established intracoronary physiological indices used to guide revascularization and risk stratification in CAD. FFR remains the gold standard for assessing the functional significance of epicardial stenoses, particularly intermediate lesions, and is recommended by the Society for Cardiovascular Angiography & Interventions and major guidelines; FFR-guided PCI improves outcomes compared with angiography alone [79,107,108]. IFR is a non-hyperemic, adenosine-free alternative measured during the diastolic wave-free period, with large trials such as DEFINE-FLAIR demonstrating noninferiority to FFR over 1–5 years, fewer procedural complications, and comparable MACE rates, making it particularly useful when adenosine is contraindicated [81,108]. IMR quantifies microvascular resistance independently of epicardial stenosis and is valuable for assessing microvascular dysfunction in acute coronary syndromes, myocardial infarction, and ANOCA, with prognostic relevance for adverse outcomes [81,82]. Patients having a working diagnosis of MINOCA showed a significant elevation in average IMR compared to control patients [109]. Identification of patients with extensive microvascular obstruction associated with STEMI is possible using invasive microvascular resistance measurements (IMR) [110].

Emerging image-based and computational approaches, including angiography-derived FFR, quantitative flow ratio (QFR), and angio-IMR, aim to reduce procedural time, cost, and complications while maintaining diagnostic and prognostic accuracy, potentially enabling broader adoption of physiology-guided strategies [83,84,85,86]. Comprehensive assessment combining FFR/iFR with IMR and absolute coronary flow measurements is being developed to better differentiate epicardial and microvascular disease, enhance patient stratification, and optimize management, with integration into risk models and decision algorithms anticipated to improve prognostic prediction in intermediate stenosis [85,86,87,88]. In summary, FFR, iFR, and IMR are central to contemporary coronary physiology assessment, and advances in noninvasive, computational, and integrated approaches are poised to expand their clinical utility [80,81,83,84,85,86,87].

For the invasive assessment of coronary microcirculation, a comprehensive evaluation combining fractional flow reserve (FFR), coronary flow reserve (CFR), and the index of microcirculatory resistance (IMR), along with acetylcholine-provocative testing, is required to confirm the diagnosis and differentiate between structural and functional microvascular disease [111].

### Practical Considerations and Integration of Intracoronary Imaging in the Assessment of Myocardial Ischemia

In contemporary coronary intervention, intravascular imaging and physiologic assessment are increasingly recommended by both European and American guidelines to optimize lesion evaluation and PCI outcomes. IVUS and OCT are endorsed for procedural guidance in complex lesions, including left main, long lesions, bifurcations, chronic total occlusions, and ambiguous culprit lesions, helping determine stent sizing, landing zones, and mechanisms of stent failure, with ESC 2023–2024 and ACC/AHA 2025 guidelines assigning Class I–IIa recommendations for their use in acute and chronic coronary syndrome settings [2,105,112]. OCT provides higher resolution and 3D reconstruction but requires contrast and has limited penetration, while IVUS penetrates deeper and is preferred in ostial left main lesions [113]. Still, it is worth mentioning that contrary to IVUS, OCT requires the usage of contrast which increases contrast burden [113]. Complementing imaging, physiologic indices guide functional decision-making: FFR assesses the hemodynamic significance of intermediate stenoses and improves outcomes versus angiography alone, though it requires hyperemic agents; iFR offers a non-hyperemic alternative with comparable efficacy in large trials; and IMR evaluates microvascular resistance, particularly after myocardial infarction or in nonobstructive coronary disease, with prognostic implications for myocardial recovery. 

FFR and iFR are preferred over CFR, PET, and CMR MBF for guiding revascularization decisions in patients with angiographically intermediate epicardial stenoses, particularly when noninvasive evidence of ischemia is absent or indeterminate [79,81,82,83,84]. In contrast, CFR, PET MBF, and CMR MBF are preferred for the evaluation of microvascular dysfunction, risk stratification, and when noninvasive assessment is needed, especially in patients with persistent symptoms and nonobstructive CAD [81,82]. IMR is reserved for invasive assessment of microvascular disease when noninvasive tests are inconclusive or when microvascular angina is suspected [81].

The choice between these modalities should be guided by clinical context, local expertise, and test availability [81,82]. For example, FFR/iFR are most appropriate when the primary question is the significance of an epicardial stenosis for revascularization, whereas PET/CMR/CFR are indicated when microvascular dysfunction or global myocardial perfusion assessment is required. 

## 10. The Summary of Practical Implications and Patient Oriented Approach to Multimodality Imaging in the Assessment of Myocardial Ischemia

At the end, when choosing between the right diagnostic tool in the assessment of myocardial ischemia, we must put into consideration availability, safety and cost. Only tertiary medical centers have advanced imaging techniques such as magnetic resonance and nuclear imaging methods. Despite the current ESC Guidelines recommendations [7], in many centers, the first step of approaching the patient with suspicion of myocardial ischemia is still simple treadmill or bicycle exercise test, which has the lowest sensitivity of 64–66% [7,8]. However, the diagnostic accuracy of different diagnostic modalities in detection of obstructive coronary disease is hard to estimate. In one of the most comprehensive systematic reviews and meta-analyses of 104 studies published from 1990 to 2025, Sonaglioni and authors showed that stress echocardiography had a sensitivity of 81%, specificity of 85%, stress SPECT had a similar sensitivity of 82%, but lower specificity of 74%, and the most superior test was stress CMR with sensitivity of 83% (dipyridamole CMR 85%), and high specificity of 89% [7]. In a different study, stress computed tomography myocardial perfusion (CTP) showed a sensitivity of 91%, with lower specificity—74. It is essential to remain aware of the radiation exposure associated with cardiac imaging modalities and to adequately inform patients of the potential risks and consequences. The estimated effective radiation dose (ERD) for a single CT coronary angiography (CCTA) protocol ranges from approximately 2 to 5 mSv, corresponding to the exposure of roughly 100 to 250 chest radiographs. Invasive coronary angiography may result in substantially higher exposure, with ERD values reaching up to 20 mSv—equivalent to nearly 1000 chest radiographs. Nuclear imaging modalities demonstrate a wider range of radiation doses: SPECT and PET protocols may vary from 2 to 20 mSv, although PET is generally considered safer, as doses typically remain below 5 mSv [114]. Clinical experience indicates that patients frequently underestimate the magnitude of radiation to which they are exposed. Bedetti et al. reported that 79% of 109 patients undergoing stress scintigraphy underestimated their radiation dose by at least 500 times, and 11% believed their exposure to be zero [115]. These findings underscore the need for clear communication regarding radiation risks. Particular attention should be given to weighing potential harms against clinical benefits, especially in vulnerable subpopulations such as women of reproductive age, younger individuals, and immunocompromised patients [116]. The economic burden of CAD is substantial, estimated at EUR 77 billion in the European Union in 2021 and USD 240 billion in the United States in 2019, with costs expected to rise further in the future [117]. Costs of diagnostic and treatment strategies vary depending on the healthcare setting, a country’s reimbursement policies, equipment availability, and staff expertise. For instance, CMR followed by CA procedure costs EUR 932 in Germany, GBP 1075 in the UK, and CHF 3252 in Switzerland [118]. Significant cost differences also exist among different diagnostic strategies for patients with suspected CAD, with total costs in the U.S. reported as CMR USD 19,273, SPECT USD 19,578, CCTA USD 19,886, and immediate CA USD 20,929. Regarding cost-effectiveness, Ge et al. reported that the CMR strategy strongly dominated both SPECT and CCTA, with an Incremental Cost-Effectiveness Ratio (ICER) of USD 52,000/Quality-Adjusted Life Year (QALY), whereas the immediate CA strategy had an ICER of USD 12 million/QALY [119]. Although advanced modalities have higher initial costs, they are economically justified due to superior diagnostic performance and reduced need for unnecessary invasive procedures, which highlights the importance of using healthcare resources efficiently, integrating advanced imaging modalities appropriately, and implementing careful risk stratification and patient triage.

## 11. The Future of Multimodal Imaging in the Assessment of Myocardial Ischemia

In the present time, when AI and machine learning models are improving rapidly, their potential is becoming increasingly evident and can be incorporated in clinical practice. The potential for accumulation and analysis of immense amounts of data provides new and valuable information. For example, one machine learning model included the data from both CCTA and stress CMR and outperformed both individual methods in predicting MACE in patients with newly diagnosed CAD [120]. The AI models can even be used to integrate and predict new markers of coronary artery disease and generate clinical prediction models [121]. More importantly, implementation of new software has facilitated the development of improved diagnostic methods that can reduce the risk of invasive complications, such as QFR—the method of evaluation of the coronary flow reserve based on a 3D angiography model, without requiring the wire pressure. Although QFR is thought to be non-inferior compared to FFR, FFR is still the preferred method in clinical decision making, especially in intermediate lesions [122]. Furthermore, CT-FFR is a noninvasive method based on the analysis of fluid dynamics techniques and machine learning algorithms, nonetheless, its predictive value remains limited, especially in cases of extensive calcifications, motion artifacts, distal or diffuse lesions, thus is not widely recommended [123].

The most comprehensive assessment of myocardial ischemia can be achieved through hybrid imaging, which combines two or more imaging modalities performed at the same time. Thus, it is possible to obtain both anatomical and functional information about CAD within a single assessment. The most commonly used hybrid imaging methods include SPECT-CT, PET-CT, and PET-MRI. Variants of hybrid imaging also include the above mentioned CT-FFR and CTP [124]. This allows for an improvement in sensitivity, spatial resolution, and quantitative accuracy, enabling not only the evaluation of myocardial ischemia but also the characterization of coronary plaque. In a large meta-analysis, hybrid cardiac imaging combining CCTA with myocardial perfusion imaging demonstrated improved diagnostic specificity for detecting obstructive CAD compared with stand-alone CCTA; however, the overall improvement in diagnostic performance was relatively modest [125].

## 12. Conclusions

In conclusion, multimodal imaging plays a pivotal role in the thorough evaluation and management of myocardial ischemia by harnessing the complementary strengths of various diagnostic techniques. Noninvasive functional imaging, including stress echocardiography, CMR, SPECT, and PET, provides valuable insights into myocardial perfusion and ischemic burden and is typically recommended for patients with intermediate to high pre-test probability of coronary artery disease, whereas coronary CTA is favored for those at low to intermediate risk. Stress echocardiography is particularly useful for identifying wall motion abnormalities and detecting multivessel or left main disease, which may be underestimated by perfusion-based modalities. CMR offers high spatial resolution along with quantitative assessment of myocardial blood flow, while SPECT and PET enable ischemic burden quantification, with PET demonstrating superior accuracy for myocardial blood flow and microvascular assessment. Invasive coronary angiography remains the definitive standard for delineating the presence, location, and severity of epicardial coronary stenoses, and it is indicated in patients with high-risk features on noninvasive imaging, refractory symptoms despite medical therapy, or inconclusive noninvasive results. Although ICA provides high-resolution anatomical detail, it cannot directly assess microvascular function, making adjunctive physiological testing coronary function assessment essential, particularly in cases of ischemia with nonobstructive coronary arteries. Due to its procedural risks, ICA should be reserved for situations in which it is expected to influence clinical management, and intravascular imaging can further enhance lesion characterization. Ultimately, the selection of imaging modalities should be individualized, taking into account patient-specific factors, comorbidities, contraindications, and the need for anatomical versus physiological information. By integrating both noninvasive and invasive approaches, clinicians can achieve a comprehensive, patient-centered strategy that optimizes the diagnosis and management of myocardial ischemia.

## Figures and Tables

**Table 1:** Advantages and Limitations of Different Diagnostic Methods in the Assessment of Myocardial Ischemia [7,8].

Different Diagnostic Method	Advantages	Limitations
Echocardiography	Regional wall motion abnormalities, CFR- perfusion reserve, LVEF, noninvasive	Suboptimal windows, suboptimal exertion, tachyarrhythmia, poor sensitivity in balanced ischemia, operator dependent
CCTA	Quantification of atherosclerosis, verification of coronary anatomy and bypass grafts	Radiation exposure, contrast nephropathy, allergies, heavy calcifications, heart rate dependent
CMR	Tissue characterization- LGE scar detection, perfusion defects, good spatial and temporal resolution, regional wall motion abnormalities, no radiation exposure, noninvasive	Time consuming, noncompatible metal devices, claustrophobia, breath holding method, heart rate dependent
SPECT and PET	Perfusion defects, myocardial viability	Radiation exposure, attenuation artefacts
ICA	Direct lesion visualization, PCI if indicated, further functional and morphological assessment	Radiation exposure, invasive, possible serious complications
